# Prevention of Allergic Sensitization and Treatment of Cow’s Milk Protein Allergy in Early Life: The Middle-East Step-Down Consensus

**DOI:** 10.3390/nu11071444

**Published:** 2019-06-26

**Authors:** Yvan Vandenplas, Bakr Al-Hussaini, Khaled Al-Mannaei, Areej Al-Sunaid, Wafaa Helmi Ayesh, Manal El-Degeir, Nevine El-Kabbany, Joseph Haddad, Aziza Hashmi, Furat Kreishan, Eslam Tawfik

**Affiliations:** 1 KidZ Health Castle, UZ Brussel, Vrijne Unversiteit Brussel, 1090 Brussels, Belgium; 2 Department of Paediatrics, King Abdulaziz University Hospital, Jeddah 22252, Saudi Arabia; 3 Department of Paediatrics, Al Salam International Hospital, Dasma 35151, Kuwait; 4 Department of Paediatric Gastroenterology, King Abdullah Specialized Children’s Hospital, Ministry of National Guard Health Affairs, Riyadh 11426, Saudi Arabia; 5 Clinical Nutrition Department, Dubai Health Authority, P.O. Box, Dubai 4545, UAE; 6 Department of Paediatrics, National Guard Hospital, Dammam 31412, Saudi Arabia; 7 Department of Paediatrics, Mediclinic Welcare Hospital, P.O. Box, Dubai 31500, UAE; 8 Department of Paediatrics, Saint George Hospital University Medical Center, Balamand University, P.O. Box, Beirut 166378, Lebanon; 9 Department of Clinical Nutrition Services, King Abdulaziz Medical City-Jeddah, Ministry of National Guard Health Affairs, Jeddah 21423, Saudi Arabia; 10 Department of Paediatrics, Alhakeem Furat Clinic, Amman 11942, Jordan; 11 Department of Paediatrics, Sheikh Khalifa Medical City, P.O. Box, Abu Dhabi 51900, UAE

**Keywords:** partial hydrolysate, cow’s milk protein allergy, hydrolysate, infant feeding, Middle-East, step-down, infant allergy

## Abstract

Allergy risk has become a significant public health issue with increasing prevalence. Exclusive breastfeeding is recommended for the first six months of life, but this recommendation is poorly adhered to in many parts of the world, including the Middle-East region, putting infants at risk of developing allergic sensitization and disorders. When breastfeeding is not possible or not adequate, a partially hydrolyzed whey formula (pHF-W) has shown proven benefits of preventing allergy, mainly atopic eczema, in children with a genetic risk. Therefore, besides stimulating breastfeeding, early identification of infants at risk for developing atopic disease and replacing commonly used formula based on intact cow milk protein (CMP) with a clinically proven pHF-W formula is of paramount importance for allergy prevention. If the child is affected by cow’s milk protein allergy (CMPA), expert guidelines recommend extensively hydrolyzed formula (eHF), or an amino acid formula (AAF) in case of severe symptoms. The Middle-East region has a unique practice of utilizing pHF-W as a step-down between eHF or AAF and intact CMP, which could be of benefit. The region is very heterogeneous with different levels of clinical practice, and as allergic disorders may be seen by healthcare professionals of different specialties with different levels of expertise, there is a great variability in preventive and treatment approaches within the region itself. During a consensus meeting, a new approach was discussed and unanimously approved by all participants, introducing the use of pHF-W in the therapeutic management of CMPA. This novel approach could be of worldwide benefit.

## 1. Introduction

Cow’s milk protein allergy (CMPA) is the most common form of food allergy in early childhood, and its prevalence has been on a steady rise over the years [1]. Intact cow milk protein (CMP) is usually the first food exposure given to an infant, and an adverse reaction to CMP is often the first symptom of an atopic condition in children [2]. CMPA in infancy is closely associated with other atopic manifestations, including 3–6 times higher risk of atopic eczema, allergic rhinitis, and asthma at 10 years of age [3].

## 2. Allergy Risk in Early Life 

A positive family history, including history of allergic disorders in parents and/or siblings, is considered to be a strong determinant of allergy risk in an infant. The risk is shown to be even higher in the case of a history of atopic eczema or asthma in the family [4]. In addition, environmental factors in the pre-, peri-, and postnatal periods also seem to influence the risk of allergies in early life (Table 1) [4]. However, a negative family history at birth does not rule out the future risk of allergy; the child is demonstrated to have similar levels of allergy risk if an immediate family member becomes allergic after the birth of the child [5].

## 3. Clinical Presentation and Diagnosis of CMPA

The first step in diagnosing CMPA involves a thorough medical history and physical examination, which can be completed with diagnostic tests to be interpreted in the context of a medical history. CMPA can induce a wide range of symptoms that could be ‘‘immediate’’ (from minutes up to 2 hours) and ‘‘delayed’’ (up to 48 hours or even 1 week) after the exposure, or a combination of both [7]. Immediate reactions are more likely to be IgE-mediated, whereas delayed reactions may also involve non-IgE-mediated immune mechanisms. Symptoms and signs of CMPA commonly involve the skin, digestive, and respiratory systems [7]. There may be an overlap between IgE-positive and IgE-negative symptomatology, especially in cases of symptoms involving the gastrointestinal system, such as allergic proctitis or proctocolitis [7]. However, certain symptoms such as angioedema and atopic eczema are relatively specific to positive CMP-specific IgE [7]. Some clinically useful diagnostic tests for CMPA are as follows:

### 3.1. IgE-Mediated 

If IgE-mediated allergy is suspected based on a focused clinical history, a skin prick test or blood tests for specific IgE antibodies to the suspected foods and likely co-allergens are indicated for diagnosis [6,7,8]. The two tests show variable sensitivity and specificity [6]. It is important to acknowledge that a positive skin prick test or a positive serum specific IgE blood test shows sensitization (i.e., presence of IgE antibodies) to a food allergen, but, on its own, does not confirm an allergy [8]. Oral challenge with cow milk protein is still considered the best confirmatory method [9].

### 3.2. Non-IgE-Mediated 

If non-IgE-mediated allergy is suspected based on the clinical history, a trial elimination of cow milk protein (normally for between 2 and 6 weeks) and reintroduction after the trial period is indicated for diagnosis [6]. The cow’s milk-related symptom score (CoMiSS) is a simple, fast, and easy-to-use awareness tool for cow’s milk-related symptoms including general, dermatological, gastrointestinal, and respiratory symptoms. However, it does not diagnose CMPA and does not replace the food challenge [10].

## 4. Allergy Prevention and CMPA Treatment in the Middle-East

### 4.1. Allergy Prevention

Exclusive breastfeeding up to 6 months of age is the preferred feeding for all infants. The World Health Organization recommends exclusive breastfeeding up to 6 months of age, with continued breastfeeding along with appropriate complementary foods up to two years of age or beyond [11]. However, the breastfeeding recommendations are poorly adhered to in the Middle-East region [12,13,14,15]. Cow-milk-based formulas are offered to infants when breastfeeding is not possible or not sufficient, placing the vulnerable children at risk of developing CMPA and at increased risk of atopic eczema. The region is also known for consuming other types of milk that have proven cross-reactivity to cow milk, including that from goat, sheep, and buffalo [16,17,18]. Camel’s milk has shown low cross-reactivity with cow milk and may be a safer alternative than other types of milk such as goat milk [19]. However, although rare, cutaneous and systemic allergic reactions to camel’s milk have been reported in the literature [20,21].

### 4.2. CMPA Treatment

The recommended management of CMPA involves strict avoidance of intact CMP, by replacing it with extensively hydrolyzed formula (eHF) or amino acid formula (AAF) in case of severe symptoms such as anaphylaxis. The diagnosis is confirmed with a positive challenge test. Later, CMP is reintroduced when tolerated after a successful challenge [7,22]. However, there is a unique approach adopted in certain institutes within the Middle-East, which involves using partially hydrolyzed whey formula (pHF-W) as a bridge between eHF or AAF and the intact CMP. There is some limited data on the benefits of oral immunotherapy in CMPA using pHF vs eHF in improving the tolerance to intact CMP [23].

The Middle-East Step-Down Consensus meeting was organized to evaluate the potential of this unique approach and provide practical recommendations to clinicians on the prevention of allergy and the management of CMPA. 

## 5. Methods

For the development of a regional consensus, 10 leading experts from the Kingdom of Saudi Arabia, UAE, Lebanon, Jordan, and Kuwait convened in a meeting. A structured quantitative method was employed to facilitate the discussion and reach a consensus [24]. Statements were prepared before the consensus meeting, based on local clinical practice and discussions with experts from the region. Before the voting, each of the statements was extensively discussed within the group and amended. All group members voted anonymously, and a nine-point scale was used to quantify the consensus (1 for strongly disagree to 9 for fully agree). A vote of 6 and above meant "agreement", and a vote of 9 was considered an expression of stronger agreement than 6. Consensus was considered to be achieved if over 75% of the votes were of the scale of “6, 7, 8, or 9”. 

## 6. Consensus Recommendations

### 6.1. Prevention

**Figure 1:**
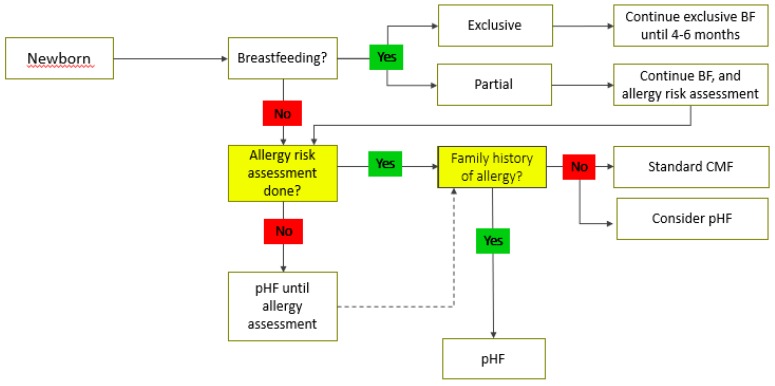
Middle-East Consensus algorithm for the prevention of allergy. BF: breastfeeding; CMF: cow milk protein; pHF: partially hydrolyzed formula.

Even though breast milk contains intact human proteins, they are most likely partially pre-digested by proteases within the human mammary gland; CMP is present as peptides. Hence the breastfed infant receives partially pre-digested proteins [25]. When breastfeeding is not possible or sufficient, certain pHF-W have shown benefits in prevention of allergy, especially atopic eczema in at-risk infants [26,27,28,29]. Animal models have shown that pHF-W is also able to induce oral tolerance, whereas extensively hydrolyzed proteins are less likely to do so [30,31]. In addition, pHF-W also offers better gastrointestinal tolerance and better digestibility compared to cow’s milk and whey- or casein-predominant standard formula with intact proteins [32,33]. However, not all pHFs have been able to demonstrate the same clinical benefits in allergy prevention [34]. Healthcare professionals should critically evaluate clinical evidence for hydrolyzed protein used in each formulation before recommending it. Goat, sheep, and buffalo milk have no indication in the prevention of atopic disease. Moreover, not all healthcare facilities have the resources to obtain an allergy risk assessment immediately at birth, and the infant should be considered to be at risk of allergy until the risk assessment has been done. The participants agreed that a clinically proven pHF-W formula can play an important role in allergy prevention (Table 2, Figure 1).

### 6.2. Treatment

In non-breastfed infants, cow-milk-based formula and supplementary foods containing CMP or other unmodified animal milk proteins such as goat milk and sheep milk should be strictly avoided [7]. An elimination diet in formula-fed infants usually involves an eHF with proven efficacy in infants with CMPA, which should be tolerated by at least 90% of children with a proven CMPA [7,35]. 

Soy protein-based formula may be an option in infants older than 6 months if an alternative to eHF is needed, provided that the tolerance to soy protein has been established. Soy contains isoflavones and phytate, which may affect nutrient absorption that makes soy not suitable under the age of 6 months [7]. The American Academy of Pediatrics acknowledges that 10 to 14% of infants with CMPA will also become allergic to soy [36]. Rice drinks are not recommended because of the high arsenic content and since they are nutritionally not adapted to the need of infants [37,38]. However, hydrolyzed rice-based infant formulae, which are nutritionally adapted and have an arsenic content similar to that of cow milk-based infant formula, are on the market in some countries [39].

When a child is put on eHF, and if there is no improvement within 2 to 4 weeks, an allergic reaction to the remaining peptides in the eHF can be considered, particularly in infants with sensitization against multiple foods. In these cases, an AAF should be tried before CMPA is ruled out as cause of the symptoms [7]. In infants with extremely severe or life-threatening symptoms, an AAF is considered as the first choice [7]. 

#### The Step-Down Approach for CMPA Treatment

**Figure 2:**
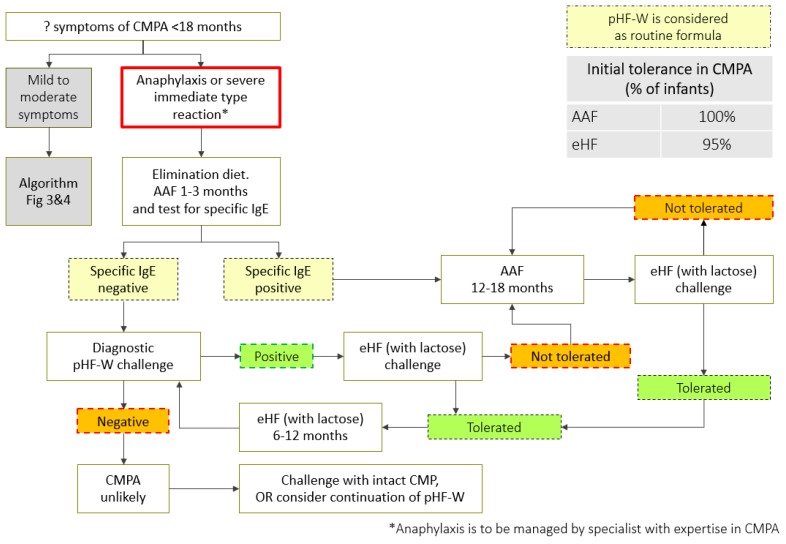
CMPA step-down treatment algorithm (anaphylaxis). AAF: amino acid based formula; eHF: extensively hydrolyzed formula; pHF-W: partially hydrolyzed formula-Whey; CMPA: cow’s milk protein allergy; CMP: cow milk protein. *Anaphylaxis is to be managed by specialist with expertise in CMPA.

**Figure 3:**
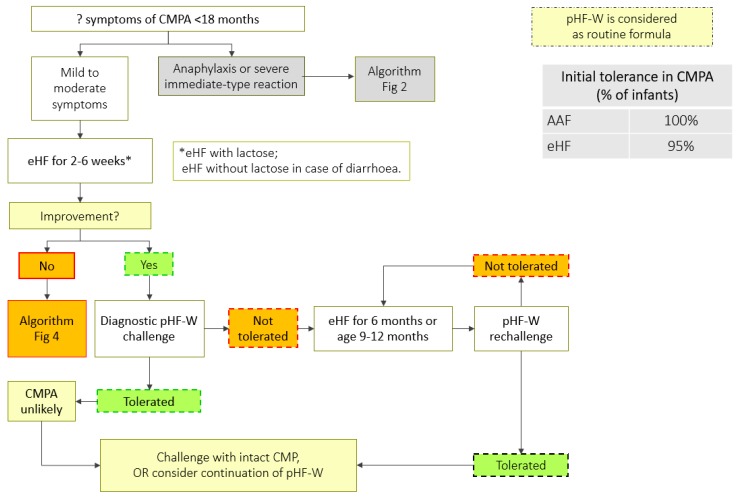
CMPA step-down treatment algorithm (mild-to-moderate symptoms). AAF: amino acid based formula; eHF: extensively hydrolyzed formula; pHF-W: partially hydrolyzed formula-Whey; CMPA: cow’s milk protein allergy; CMP: cow milk protein. * use eHF without lactose in case of diarrhoea.

**Figure 4:**
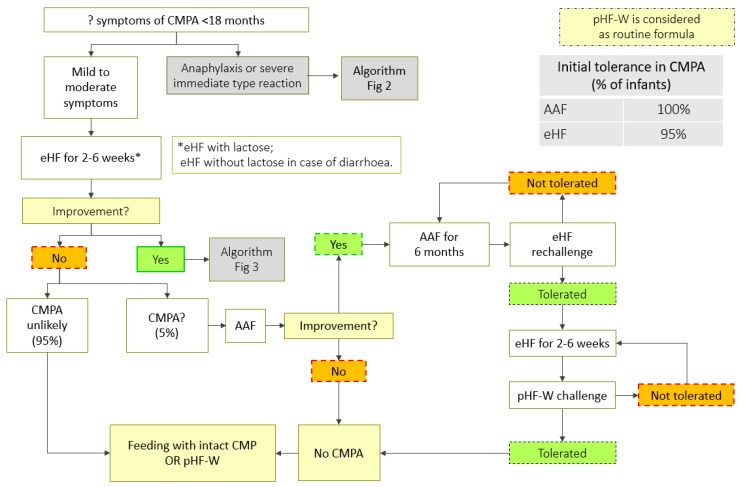
CMPA step-down treatment algorithm (mild-to-moderate symptoms). AAF: amino acid based formula; eHF: extensively hydrolyzed formula; pHF-W: partially hydrolyzed formula-Whey; CMPA: cow’s milk protein allergy; CMP: cow milk protein. * use eHF without lactose in case of diarrhoea.

A step-down approach can be considered while managing children with CMPA, using pHF-W as a bridge between eHF or AAF and the intact CMP (Table 3, Figure 2, Figure 3 and Figure 4). However, strict protocols need to be adhered to, including a carefully conducted pHF-W challenge, before initiating this approach (Table 4).

Open oral challenge is usually the first step especially in low-risk groups. A standardized oral challenge test is performed under medical supervision. A double-blind, placebo-controlled food challenge is the reference standard and the most specific test; however, the test is time-consuming and expensive. Therefore, an open oral challenge is usually the first step, particularly if the history indicates a low likelihood of a reaction. The oral challenge should be performed with an infant formula based on cow milk in the first year of life [7]. 

## 7. Discussion

This step-down protocol for CMPA management includes the use of pHF-W, considered as an alternative standard infant formula. Although evidence from the literature is not available, it is well known that many healthcare professionals advice the use of pHF-W when CMPA is suspected. Many HCPs recommend “hydrolysates” in the management of infants suspected to suffer CMPA, but mix partial and extensive hydrolysates. A European survey discovered major deficits in the management of CMPA, including limited knowledge of diagnostic tests, eliminations, and selection of formula for the management of CMPA in non-breastfed infants [40]. 

All existing guidelines recommend eHF in the management of CMPA, but none mention pHF-W in the algorithm. In this algorithm, we propose pHF as an alternative to standard infant formula with intact protein, as we recommend pHF-W as the formula to be used in the challenge test (after strong improvement or disappearance of the symptoms with 2–4 weeks of eHF). We hypothesize that this algorithm will result in a decrease in the misuse of pHF in the treatment of CMPA. However, non-IgE mediated allergy might be difficult to distinguish from functional gastro-intestinal disorders. Nutritional treatment with pHF-W is a recommended approach in the management of functional gastro-intestinal disorders [41]. Complete elimination of lactose from the infant’s diet is disadvantageous for the development of a healthy gut microbiota and does result in a decreased calcium absorption [42,43,44].

An allergy-focused clinical history is a must before carrying out allergy testing. The skin and blood tests as well as oral challenge should be undertaken by healthcare professionals with appropriate competencies to select, perform, and interpret them. The oral challenge should be undertaken under medical supervision, and in an inpatient setting in case a severe reaction could occur [6]. The role of soy-based formula in treatment of CMPA is still debated, but is not recommended below 6 months of age [7]. Goat, sheep, and buffalo milk are not suitable alternatives for CMPA prevention or treatment because they are not nutritionally adapted (if not marketed as infant formula) and cross-react with cow milk protein [45]. 

## 8. Conclusions

The Middle-East Step-Down approach of managing CMPA using pHF-W as a bridge between eHF or AAF and intact CMP has great potential to improve the lives of affected children and families. This uncovers another clinically relevant facet of a specific pHF-W that was shown to prevent atopic eczema in children at risk of allergy. However, clinicians should keep in mind that not all pHFs are the same and choose the pHF-W formula according to published evidence.

## Figures and Tables

**Table 1:** Risk factors for allergy [4,6].

Family history Environmental factors Formula feeding (with intact protein) Shorter duration of breastfeeding Older maternal age Higher parity Prematurity Caesarean delivery

**Table 2:** Middle-East Consensus statements on the prevention of allergy.

Sr. No.	Statement	Agreement
1	Exclusive breastfeeding up to 6 months is the best feeding for every infant to achieve optimal growth, development, and health (WHO statement).	100% (rating 9)
2	When breastfeeding is not possible or when breast milk is not available, partially hydrolyzed whey formula (pHF-W) with documented safety and efficacy should be recommended for infants at risk of allergy.	100% (rating 9)
3	Not all pHFs are the same, as different formulations have different peptide compositions and production methods and have demonstrated different outcomes.	100% (rating 9)
4	When breastfeeding is not possible or when breast milk is not available, pHF-W with documented safety and efficacy could be considered for all infants.	100% (rating 9)

**Table 3:** Consensus statements on cow’s milk protein allergy (CMPA) treatment: The Middle-East step-down approach.

Sr. No.	Statement	Agreement
1	Management of cow milk protein allergy involves avoidance of cow milk protein, through extensively hydrolyzed formula (eHF) in most of the infants, or if it is not tolerated, amino acid formula (AAF).	100% (rating 9)
2	In case of anaphylaxis, start with AA-based formula	100% (rating 9)
3	pHF-W can be used in the transition from eHF or AAF to intact CMP, if the initial pHF-W challenge is tolerated by the child.	100% (rating 9)
4	pHF formulas should not be interchanged, as the formulas differ in their clinical outcomes.	100% (rating 9)

**Table 4:** Oral challenge protocol [7].

Place a drop of milk on the inside of lower lip and observe for reaction; if no reaction, the dose can be increased every 30 minutes until 100 mL If severe reactions expected: Stepwise dosing of 0.1, 0.3, 1.0, 3.0, 10.0, 30.0, and 100 mL given at 30-minute intervals If delayed reactions expected: Stepwise dosing of 1, 3.0, 10.0, 30.0, and 100 mL given at 30-minute intervals Patients should be observed for at least 2 hours following the maximum dose If no reaction, then the milk should be continued at home every day with at least 200 mL/day for at least 2 weeks Parents should be contacted by telephone to document any potential late reactions

## References

[1] Savage J., Johns C.B. (2015). Food allergy: Epidemiology and natural history. Immunol. Allergy Clin. North Am..

[2] Oranje A.P., Wolkerstorfer A., de Waard-van der Spek F.B. (2002). Natural course of cow’s milk allergy in childhood atopic eczema/dermatitis syndrome. Ann. Allergy Asthma Immunol..

[3] Tikkanen S., Kokkonen J., Juntti H., Niinimäki A. (2000). Status of children with cow’s milk allergy in infancy by 10 years of age. Acta Paediatr..

[4] Sardecka I., Łoś-Rycharska E., Ludwig H., Gawryjołek J., Krogulska A. (2018). Early risk factors for cow’s milk allergy in children in the first year of life. Allergy Asthma Proc..

[5] Fuertes E., Standl M., von Berg A., Lehmann I., Hoffmann B., Bauer C.-P., Koletzko S., Berdel D., Heinrich J. (2015). Parental allergic disease before and after child birth poses similar risk for childhood allergies. Allergy.

[6] National Institute for Health and Clinical Excellence (2011). Food Allergy in Children and Young People.

[7] Koletzko S., Niggemann B., Arato A., Dias J.A., Heuschkel R., Husby S., Mearin M.L., Papadopoulou A., Ruemmele F.M., Staiano A. (2012). Diagnostic approach and management of cow’s-milk protein allergy in infants and children: ESPGHAN GI Committee practical guidelines. J. Pediatr. Gastroenterol. Nutr..

[8] Venter C., Brown T., Meyer R., Walsh J., Shah N., Nowak-Węgrzyn A., Chen T.-X., Fleischer D.M., Heine R.G., Levin M. (2017). Better recognition, diagnosis and management of non-IgE-mediated cow’s milk allergy in infancy: iMAP—An international interpretation of the MAP (Milk Allergy in Primary Care) guideline. Clin. Transl. Allergy.

[9] Costa A.J.F., Sarinho E.S.C., Motta M.E.F.A., Gomes P.N., de Oliveira de Melo S.M., da Silva G.A.P. (2011). Allergy to cow’s milk proteins: What contribution does hypersensitivity in skin tests have to this diagnosis?. Pediatr. Allergy Immunol..

[10] Vandenplas Y., Dupont C., Eigenmann P., Host A., Kuitunen M., Ribes-Koninckx C., Shah N., Shamir R., Staiano A., Szajewska H. (2015). A workshop report on the development of the Cow’s Milk-related Symptom Score awareness tool for young children. Acta Paediatr..

[11] Breastfeeding. https://www.who.int/topics/breastfeeding/en/.

[12] Alfaleh K., Alluwaimi E., Aljefri S., Alosaimi A., Behaisi M. (2014). Infant formula in saudi arabia: A cross sectional survey. Kuwait Med. J..

[13] Al-Nuaimi N., Katende G., Arulappan J. (2017). Breastfeeding Trends and Determinants: Implications and recommendations for Gulf Cooperation Council countries. Sultan Qaboos Univ. Med. J..

[14] Alzaheb R.A. (2017). A review of the factors associated with the timely initiation of breastfeeding and exclusive breastfeeding in the Middle East. Clin. Med. Insights Pediatr..

[15] Gardner H., Green K., Gardner A. (2015). Infant feeding practices of emirati women in the rapidly developing city of Abu Dhabi, United Arab Emirates. Int. J. Environ. Res. Public Health.

[16] Sheehan W.J., Gardynski A., Phipatanakul W. (2009). Skin testing with water buffalo’s milk in children with cow’s milk allergy. Pediatr. Asthma. Allergy Immunol..

[17] Restani P., Beretta B., Fiocchi A., Ballabio C., Galli C.L. (2002). Cross-reactivity between mammalian proteins. Ann. Allergy Asthma Immunol..

[18] Bellioni-Businco B., Paganelli R., Lucenti P., Giampietro P.G., Perborn H., Businco L. (1999). Allergenicity of goat’s milk in children with cow’s milk allergy. J. Allergy Clin. Immunol..

[19] Ehlayel M., Bener A., Abu Hazeima K., Al-Mesaifri F. (2011). Camel milk is a safer choice than goat milk for feeding children with cow milk allergy. ISRN Allergy.

[20] Ehlayel M., Bener A. (2018). Camel’s milk allergy. Allergy Asthma Proc..

[21] Al-Hammadi S., El-Hassan T., Al-Reyami L. (2010). Anaphylaxis to camel milk in an atopic child. Allergy.

[22] Vandenplas Y., Abuabat A., Al-Hammadi S., Aly G.S., Miqdady M.S., Shaaban S.Y., Torbey P.-H. (2014). Middle east consensus statement on the prevention, diagnosis, and management of cow’s milk protein allergy. Pediatr. Gastroenterol. Hepatol. Nutr..

[23] Inuo C., Tanaka K., Suzuki S., Nakajima Y., Yamawaki K., Tsuge I., Urisu A., Kondo Y. (2018). Oral immunotherapy using partially hydrolyzed formula for cow’s milk protein allergy: A randomized, controlled trial. Int. Arch. Allergy Immunol..

[24] CDC Evaluation Research Team Gaining Consensus among Stakeholders Through the Nominal Group Technique. https://www.cdc.gov/healthyyouth/evaluation/pdf/brief7.pdf.

[25] Nielsen S.D., Beverly R.L., Dallas D.C. (2017). Milk proteins are predigested within the human mammary gland. J. Mammary Gland Biol. Neoplasia.

[26] von Berg A., Filipiak-Pittroff B., Krämer U., Hoffmann B., Link E., Beckmann C., Hoffmann U., Reinhardt D., Grübl A., Heinrich J. (2013). Allergies in high-risk schoolchildren after early intervention with cow’s milk protein hydrolysates: 10-year results from the German Infant Nutritional Intervention (GINI) study. J. Allergy Clin. Immunol..

[27] von Berg A., Koletzko S., Grübl A., Filipiak-Pittroff B., Wichmann H.-E., Bauer C.P., Reinhardt D., Berdel D., German Infant Nutritional Intervention Study Group (2003). The effect of hydrolyzed cow’s milk formula for allergy prevention in the first year of life: the German Infant Nutritional Intervention Study, a randomized double-blind trial. J. Allergy Clin. Immunol..

[28] von Berg A., Filipiak-Pittroff B., Schulz H., Hoffmann U., Link E., Sußmann M., Schnappinger M., Brüske I., Standl M., Krämer U. (2016). Allergic manifestation 15 years after early intervention with hydrolyzed formulas--the GINI Study. Allergy.

[29] Szajewska H., Horvath A. (2017). A partially hydrolyzed 100% whey formula and the risk of eczema and any allergy: an updated meta-analysis. World Allergy Organ. J..

[30] Pecquet S., Bovetto L., Maynard F., Fritsché R. (2000). Peptides obtained by tryptic hydrolysis of bovine β-lactoglobulin induce specific oral tolerance in mice. J. Allergy Clin. Immunol..

[31] Fritsché R., Pahud J.J., Pecquet S., Pfeifer A. (1997). Induction of systemic immunologic tolerance to beta-lactoglobulin by oral administration of a whey protein hydrolysate. J. Allergy Clin. Immunol..

[32] Billeaud C., Guillet J., Sandler B. (1990). Gastric emptying in infants with or without gastro-oesophageal reflux according to the type of milk. Eur. J. Clin. Nutr..

[33] Exl B.M., Deland U., Secretin M.C., Preysch U., Wall M., Shmerling D.H. (2000). Improved general health status in an unselected infant population following an allergen-reduced dietary intervention programme: the ZUFF-STUDY-PROGRAMME. Part II: Infant growth and health status to age 6 months. ZUg-FrauenFeld. Eur. J. Nutr..

[34] Boyle R.J., Ierodiakonou D., Khan T., Chivinge J., Robinson Z., Geoghegan N., Jarrold K., Afxentiou T., Reeves T., Cunha S. (2016). Hydrolysed formula and risk of allergic or autoimmune disease: Systematic review and meta-analysis. BMJ.

[35] Giampietro P.G., Kjellman N.I., Oldaeus G., Wouters-Wesseling W., Businco L. (2001). Hypoallergenicity of an extensively hydrolyzed whey formula. Pediatr. Allergy Immunol..

[36] Bhatia J., Greer F., American Academy of Pediatrics Committee on Nutrition (2008). Use of soy protein-based formulas in infant feeding. Pediatrics.

[37] Dennis S., Fitzpatrick S., Egan K., Flannery B., Kanwal R., Smegal D., Spungen J., Tao S. (2016). Arsenic in Rice and Rice Products Risk Assessment Report.

[38] Hojsak I., Braegger C., Bronsky J., Campoy C., Colomb V., Decsi T., Domellöf M., Fewtrell M., Mis N.F., Mihatsch W. (2015). Arsenic in rice: A cause for concern. J. Pediatr. Gastroenterol. Nutr..

[39] Meyer R., Carey M.P., Turner P.J., Meharg A.A. (2018). Low inorganic arsenic in hydrolyzed rice formula used for cow’s milk protein allergy. Pediatr. Allergy Immunol..

[40] Werkstetter K., Chmielewska A., Dolinšek J., Burk F.E., Korponay-Szabó I., Kurppa K., Mišak Z., Papadopoulou A., Popp A., Ribes-Konickx C. (2018). Diagnosis and management of cow’s milk protein allergy—How big is the gap between ideal and reality? A quality-of-care survey in Europe. J. Pediatr. Gastroenterol. Nutr..

[41] Salvatore S., Abkari A., Cai W., Catto-Smith A., Cruchet S., Gottrand F., Hegar B., Lifschitz C., Ludwig T., Shah N. (2018). Review shows that parental reassurance and nutritional advice help to optimise the management of functional gastrointestinal disorders in infants. Acta Paediatr..

[42] Francavilla R., Calasso M., Calace L., Siragusa S., Ndagijimana M., Vernocchi P., Brunetti L., Mancino G., Tedeschi G., Guerzoni E. (2012). Effect of lactose on gut microbiota and metabolome of infants with cow’s milk allergy. Pediatr. Allergy Immunol..

[43] Vandenplas Y. (2015). Lactose intolerance. Asia Pac. J. Clin. Nutr..

[44] Heine R.G., AlRefaee F., Bachina P., De Leon J.C., Geng L., Gong S., Madrazo J.A., Ngamphaiboon J., Ong C., Rogacion J.M. (2017). Lactose intolerance and gastrointestinal cow’s milk allergy in infants and children—Common misconceptions revisited. World Allergy Organ. J..

[45] Järvinen K.M., Chatchatee P. (2009). Mammalian milk allergy: Clinical suspicion, cross-reactivities and diagnosis. Curr. Opin. Allergy Clin. Immunol..

